# Leveraging Contextual Sentences for Text Classification by Using a Neural Attention Model

**DOI:** 10.1155/2019/8320316

**Published:** 2019-08-01

**Authors:** DanFeng Yan, Shiyao Guo

**Affiliations:** Beijing University of Posts and Telecommunications, State Key Laboratory of Networking and Switching Technology, Beijing, China

## Abstract

We explored several approaches to incorporate context information in the deep learning framework for text classification, including designing different attention mechanisms based on different neural network and extracting some additional features from text by traditional methods as the part of representation. We propose two kinds of classification algorithms: one is based on convolutional neural network fusing context information and the other is based on bidirectional long and short time memory network. We integrate the context information into the final feature representation by designing attention structures at sentence level and word level, which increases the diversity of feature information. Our experimental results on two datasets validate the advantages of the two models in terms of time efficiency and accuracy compared to the different models with fundamental AM architectures.

## 1. Introduction

Text classification should be the most common application in natural language processing (NLP), such as automatic article classification, automatic mail classification, spam recognition, and user emotion classification [1, 2]. Therefore, it has attracted considerable attention from many researchers. After the 1990s, the number of Internet online texts and the rise of machine learning disciplines gradually formed a set of classic solutions to solve large-scale text classification problems. The main routines at this stage were artificial feature engineering + shallow classification model.

Feature engineering is divided into three parts: text preprocessing, feature extraction, and text representation, and its ultimate goal is to convert the text into a computer comprehensible format and encapsulate enough information for classification. Among them, text representation is the most important part of determining the quality of text classification. Traditional methods commonly used bag of words (BOW) [3]. However, this method suffers from high latitude and high sparsity [4, 5]. BOW model usually reduces the dimension by feature item selection, that is, the original feature items (terms) are ranked according to an evaluation index independently [6].

In addition, the density is increased by feature weight calculation which is mainly based on the classical term frequency-inverse document frequency (TF-IDF) method and its extension method [7]. The main idea is that the importance of a word is proportional to the frequency of words within the category and inversely proportional to the number of occurrences of all categories. After getting the text representation, specifying a text classifier such as logistic regression and support vector machines to train a model is necessary [8, 9]. Neglecting context and unable to express semantic information and independence between words are its biggest drawbacks of the traditional method.

Deep learning has achieved a great success in image and speech. One important reason is that image and speech raw data are not only continuous but also dense with local correlation. For now, applying deep learning to solve large-scale text classification problem is the most important thing in text representation domain. The way is to use convolutional neural network (CNN) or recurrent neural network (RNN) and other network structures to automatically acquire feature expression capabilities, remove complicated artificial feature engineering, and then solve problems end to end [10, 11]. Besides, as a most successful concept, distributed representation's basic idea is to express each word as an n-dimensional dense, continuous real vector, and it helps learning algorithms achieve better performance.

CNN recently achieved very successful results in many domains. In NLP, CNN utilizes 1D convolution to perform the feature mapping and then applies 1D max pooling operation over the time-step dimension to obtain a fixed-length output as a representation of sentence [10, 12]. RNN can capitalize on distributed representations of words by first converting the tokens comprising each text into vectors, which can form a matrix [13]. This matrix includes two dimensions: the time-step dimension and the features vector dimension. Then, the model can utilize 1D max pooling or attention-based operation, which extracts the maximum values or generates a weighted representation over the time step of the matrix, to obtain a fixed-length vector [12, 14, 15].

In deep learning works, most text classification methods based on CNN or RNN do not consider the context of sentence. However, if a sentence is given without any context, it is not always obvious even for human beings to find the corresponding category. Hence, it is necessary to take advantage of the context or sequence information to help classify the current sentence. For example, in the dataset of dialog act (DA) classification task, if the preceding utterance is a question, then the next is most likely be an answer. Such context information has been explored in some preceding methods, for example, hidden Markov models (HMM), conditional random fields (CRF), and so on [16, 17]. The main idea of leveraging contextual information is to extend the input sentences into a sequence centering on the current sentence which is to be classified.

Nevertheless, contextual sentences and current sentence should have different importance under different circumstances in classification task; otherwise, it may increase the loss of current sentence information. Obviously, attention mechanism (AM) is very suitable for solving this problem. Inspired by the performance of AMs, we explore ways using AMs in different levels in models based on CNN and LSTM to eliminate invalid information and get more accurate contextual interaction information and finally to improve performance.

Above all, this paper explores the use of AMs to learn the context representation, as a manner to fuse the context as well as highlight the critical information, ignoring unimportant parts for text classification. Especially, we design a feature selection architecture to get additional features concatenating with the representation as the input of higher level multilayer perceptron. Therefore, our contributions are summarized below:

1. This paper proposes models which leverage contextual sentences for text classification. We design two kinds of AMs to achieve the models, in which one considering from the sentence level is based on CNN simple but less accurate and the other considering from word level is based on LSTM complex but more effective and innovative.
2. In order to retain both the original features and the high-order features, we combine neural network layers with traditional methods in this paper. That is, we get the final features representation by artificial construction and feature extraction and semantic features through neural network.
3. Finally, we use time and accuracy to evaluate the proposed models and analyze their respective advantages from the results on the dialog act classification task. A dialog act characterizes an utterance in a dialog based on a combination of pragmatic, semantic, and syntactic criteria. What is crucial is that the dialog act is sequential and contextual. The experimental results indicate that our approach has a good classification performance in terms of time and accuracy.

## 2. Related Work

### 2.1. Traditional Methods

Text classification is significant for NLP systems; also, there has been a large body of research on this task. A simple and efficient method for text classification is to represent the sentence by BOW and then to train a linear classifier. There are some feature selection methods to reduce the dimension of BOW; that is, some feature items with the highest score are selected, and the remaining feature items are filtered out [6, 18, 19]. The common feature selection algorithms include chi-square test (CHI), odds ratio (OR), and Gini index (GINI) [13]. However, the most fundamental problem is that BOW ignores information about the order of words and semantics. Under this circumstance, the N-gram model is another popular way of expressing sentences, and this method usually performs the best. Words or characters are cast to a low dimensional space, and these embeddings are merged to get a fixed size representation of the input sentence and then as input of classifier. Nevertheless, an N-gram model still suffers from the data sparsity problem [20].

### 2.2. Deep Learning Methods

Deep learning-based neural network models have achieved great improvement on text classification tasks. Recently, deep neural networks and representation learning have led to new ideas for solving data sparsity problem, and many neural models for learning word representation have been proposed [21, 22]. Consequently, several neural network-based methods learn phrase and sentence representations following these approaches.

RNN's main idea is to combine the data of the previous hidden unit and the data of the input unit to achieve a “loop” effect recursively. However, this structure makes RNN more suitable for processing short-sequence data than long-sequence data because it will cause gradient explosion or gradient disappearing [23]. The long and short time memory (LSTM) network, as an extension of the RNN, solves this problem by introducing the concept of a gate [24]. Tang et al. [25] developed target-dependent LSTM where target information is automatically taken into account. Tai et al. [23] explored LSTM to Tree-LSTM, where each LSTM unit gains information from its children units. For some tasks, it would be efficient if we could take both words from the left (past) to the right (future) into consideration when making a prediction. That is exactly what the bidirectional long and short time memory (BLSTM) network does, and it consists of two LSTMs. One layer runs forward from left to right and the other runs backward from right to left [26]. This structure is also used in this paper.

A rather shallow neural net based on CNN for sentence classification was proposed in [10]: one convolutional layer with multiple widths and filters followed by a max pooling layer over time. The final classifier uses one fully connected layer with dropout. Kalchbrenner et al. [27] proposed a similar system which used deeper layers. An import difference is that a dynamic *k*-max pooling mechanism is introduced. This allows the detection of the *k* most important features in a sentence, regardless of their specific location maintaining their relative order. To explore the effect of the architecture components on performance, Zhang and Wallace [28] made a sensitivity analysis of on layer CNN and Yin and Schutze [29] introduced multichannel embeddings and unsupervised pretraining to improve classification accuracy. The combination of recurrent and convolutional networks in one architecture has also been investigated. Zhou et al. [30] trained a model using CNN to get high-level features of text which servers as the input for one layer of LSTM.

In the improvement of text analysis, the AM that was first applied to image processing has also been gradually used in the field of natural language processing. The first work in NLP to use AMs is to solve neural network machine translation (NMT) by Bahdanau et al. [18]. Luong and Manning [31] wrote a paper which is a representative paper following the previous paper. Their work told us how attention can be extended in the RNN. In the paper, they proposed two kinds of attention mechanisms, one is a global mechanism and the other is a local mechanism. A based hierarchical attention network for document classification was proposed that performs an attention first on the sentences in the document and on the words in the sentence [32]. Yin et al. [33] proposed three methods for using attention in CNN, which is an early exploratory work of attention in CNN. A relatively complex AM is introduced by the bidirectional attention flow network, which is a hierarchical multistage architecture for modeling the representations of the contextual paragraph at different levels of granularity [34]. In this paper, we also borrowed this idea when designing the attention mechanism based on LSTM.

The task of DA classification is to attribute one type of a predefined DA to each given utterance. Therefore, DA classification is also treated as text classification. Few papers based on deep learning methods have suggested to utilize context as a potential knowledge to help sentence classification. Kalchbrenner and Blunsom [35] used a mixture of CNN and RNN model on DA datasets. CNNs were used to extract local features from each utterance, and RNNs were used to create a general view of the whole dialog. Lee and Dernoncourt [36] proposed a model consisting of two parts. The first part using CNN or RNN generated a vector for one sentence; the second part classified the current sentence as well as took advantage of a few contexts.

## 3. Model

Our model is a hierarchical multistage process and consists of four layers. The first layer is word embedding layer which maps each word to a vector space using a pretrained word embedding. The second layer is representation layer which utilizes either CNN to get a vector or LSTM to get a matrix to represent each sentence and then uses different attention methods coupling the context and current sentence representation to produce an optimal representation about the sentences. The third layer is additional features layer which explores the use of traditional methods to extract features as part of the final representation. The fourth layer is output layer which provides the result of sentence classification.

### 3.1. Word Embedding Layer

Word embedding layer is responsible for mapping each word to a high-dimensional vector space which can capture semantic and syntactic information of words. Each column of the matrix stores a word embedding of the corresponding word. Let {*x*_1_ … *x*_*T*_} represent the word sequence in an input sentence. First, we use pretrained word vectors by word2vec [21] to obtain the fixed word embedding of each word, and then we fine-tune these vectors in training. Through this layer, the sentence is represented as a matrix: *X* ∈ *R*^*m*×*T*^, among which *m* is the dimension of a word vector and *T* is the length of the sentence.

### 3.2. Representation Layer

**Figure 1:**
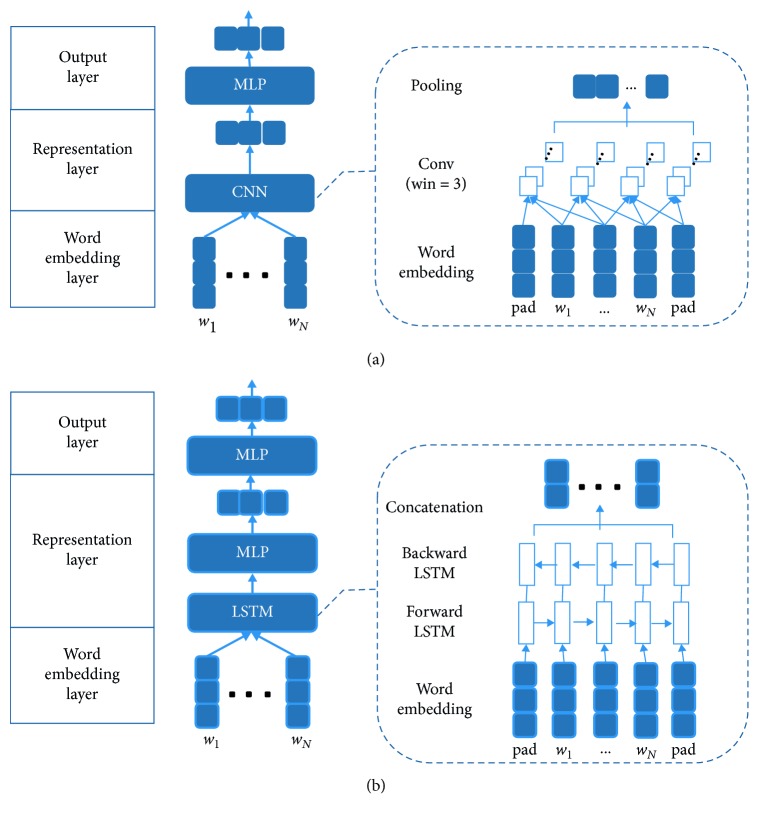
The two basic models in our experiments. One is based on CNN and the other is based on BLSTM. (a) The basic model based on CNN. The width of convolution filters is 3, and the method of pooling is max pooling. (b) The basic model based on LSTM. The output of LSTM network is a matrix concatenating the hidden vectors from forward LSTM and backward LSTM.

In this module, we will first discuss how the CNN/LSTM achieves single sentence representation and then explore the representation method of the complex model considering context using AM-based CNN/LSTM. Just as shown in Figure 1, we use a CNN network or LSTM network on top of the embedding provided by the previous layer to model a sentence representation.

#### 3.2.1. CNN-Based Representation

In text classification task, combining CNN with multilayer perception (MLP) to learn a representation is a common method so that we get our basic model by using this method as shown in Figure 1(a). Since we want to use the context to classify the current sentence, the input in our model should be multiple consecutive sentences. First, we use CNN to get the representation of each sentence which performs a discrete convolution on an input matrix with a set of different filters. A convolution operation involves a filter *W* ∈ *R*^*k*×*m*^, which is applied to a window of *k* words to produce a new feature. For example, a feature started from the *t*-th word is

(1) $$\begin{array}{c} f_{t}=f\left(w\cdot x_{t:t+k-1}+b\right), \end{array}$$

where *b* ∈ *R* is a bias term to learn, · represents matrix multiplication, and *f* is a nonlinear activation function such as rectified linear unit (RELU) which allows the network to introduce sparsity on its own while greatly improving training speed. This operation is applied to each possible window of words in the sentence to produce a feature map *c*=[*c*_1_, *c*_2_,…, *c*_*t*−*k*+1_], in which *c* ∈ *R*^*T*−*k*+1^. To simplify the model, we use only one size convolution core. Then, we apply a max pooling operation over the feature map to capture the most importance feature. Furthermore, *n* different filters will be used to perform convolution operation and all feature maps are concatenated to one vector *s* ∈ *R*^*n*^, which can be the representation of the sentence.

**Figure 2:**
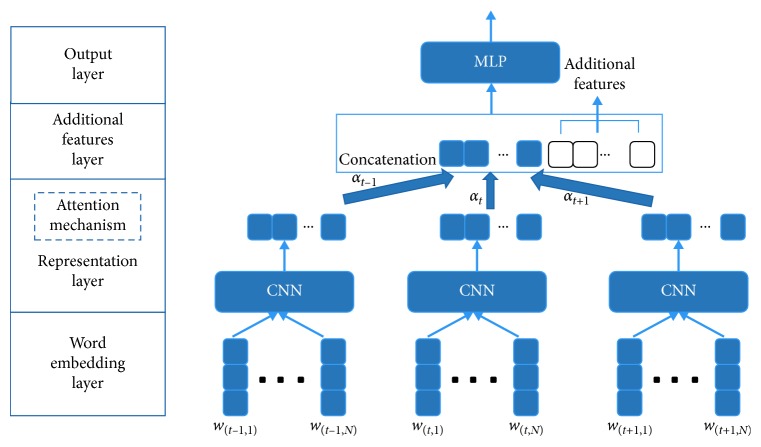
Text classification model with attention mechanism and additional features based on CNNs and consecutive sentences.

*(1) CNN-Based Attention Mechanism*. Contextual sentences can provide language environment for the current sentence to be categorized when the sentences are not independent and can be used to analyze the causal, temporal, and inheritance relationships. Obviously, these relationships can provide additional useful information for the current sentence and facilitate the judgment of the category of the current sentence. Since our goal is to find a way to integrate contextual information very well, a simpler approach is to use the attention mechanism from the sentence level to filter unwanted information and only retain important information. Let *u*_*t*_ be the *n*-dimensional representation given by the CNN architecture for the *t*-th utterance. We use AM for the consecutive utterances *u*_*t*−*n*:*t*+*m*_ representation learning as shown in Figure 2. In this module, for each of the input vector *u*_*t*−*i*_ at time step *t* − *i* in a dialog, *t* is the current time step and the attention weight *α*_*t*_ is computed as follows:

(2) $$\begin{array}{c} \alpha_{t}=\text{softmax}\left(u_{t}\right)=\frac{\exp\left(f\left(u_{t}\right)\right)}{\sum_{-n<j<m}\exp\left(f\left(u_{t+j}\right)\right)}, \end{array}$$

where *f* is the scoring function, *W* ∈ *R*^*nxn*^, *c* ∈ *R*^*n*^, and *c* is a hidden vector to be learned. To some extent, the learning result *f*(*u*_*t*_) is equivalent to the high-level semantic understanding of the initial input sentence, that is, attention weight. In order to facilitate subsequent processing and calculation, standardized operation is needed to convert it into probabilistic form. Relevantly, the softmax function takes an unnormalized vector and normalizes it into a probability distribution. That is, prior to applying softmax, some vector elements could be negative or greater than one and might not sum to 1, but after applying softmax, each element *x*_*i*_ is in the interval [0, 1], and ∑_*i*_*x*_*i*_=1.

(3) $$\begin{array}{c} f\left(u_{t}\right)=u_{t}^{T}Wc. \end{array}$$

The output *u* of representation layer is the weighted sum of the input sequences. Here, *∗* represents elementwise multiplication; that is, when scalar and nonscalar are multiplied, each element in nonscalar output is the product of corresponding elements in scalar input and nonscalar input.

(4) $$\begin{array}{c} u=\underset{-n<j<m}{\sum }\alpha_{t+j}*u_{t+j}. \end{array}$$

The other method of getting the sequence representation is to splice the weighted inputs into a vector *v* which preserves the order information.

(5) $$\begin{array}{c} v=\left[\ldots ,\alpha_{-1}u_{t-1},\alpha_{0}u_{t},\alpha_{1}u_{t+1},\ldots \right]. \end{array}$$

In this word, similar results can be obtained in the different ways.

#### 3.2.2. LSTM-Based Representation

LSTM was firstly proposed by Schuster and Paliwal [26] to overcome the gradient vanishing problem of RNN. The main idea is to introduce an adaptive gate mechanism that determines the extent to which the previous state is maintained and remembers the extracted features of the current data input. So, we use LSTM network in representation layer to model the temporal interactions between words which can solve long-term dependencies. We place an LSTM in both directions and concatenate the outputs of the two LSTM layers. Hence, we obtain *H* ∈ *R*^2*d*×*T*^ from the word vectors *X*. Note that each column of *H* is two-dimensional because of the concatenation of the outputs of the forward and backward LSTM, each with d-dimensional output. For the *t*-th word in sentence, an LSTM takes as input *x*_*t*_, *h*_*t*−1_, *c*_*t*−1_ and produces *h*_*t*_, *c*_*t*_ based on following formulas:

(6) $$\begin{array}{c} i_{t}=\sigma \left(W_{i}x_{t}+U_{i}h_{t-1}+b_{i}\right),\\ f_{t}=\sigma \left(W_{f}x_{t}+U_{f}h_{t-1}+b_{f}\right),\\ {\tilde{c}}_{t}=\tanh\left(W_{c}x_{t}+U_{c}h_{t-1}+b_{c}\right),\\ c_{t}=f_{t}\cdot c_{t-1}+i_{t}\cdot {\tilde{c}}_{t},\\ o_{t}=\sigma \left(W_{o}x_{t}+U_{o}h_{t}-1+b_{o}\right),\\ h_{t}=o_{t}\cdot \tanh\left(c_{t}\right), \end{array}$$

where *x*_*t*_ is the input at the current time step; *W*_*j*_ ∈ *R*^*T*×*d*^, *U*_*j*_ ∈ *R*^*d*×*d*^ are weight matrices; *b*_*j*_ ∈ *R*^*d*^ are bias vectors; *i*, *f*, and *o* are the input gate activation, forget gate activation, and output gate activation; $\tilde{c}$ is the current cell state; the symbols *σ*(·) and tanh(·) refer to sigmoid activation function and hyperbolic tangent function; and · denotes matrix multiplication.

**Figure 3:**
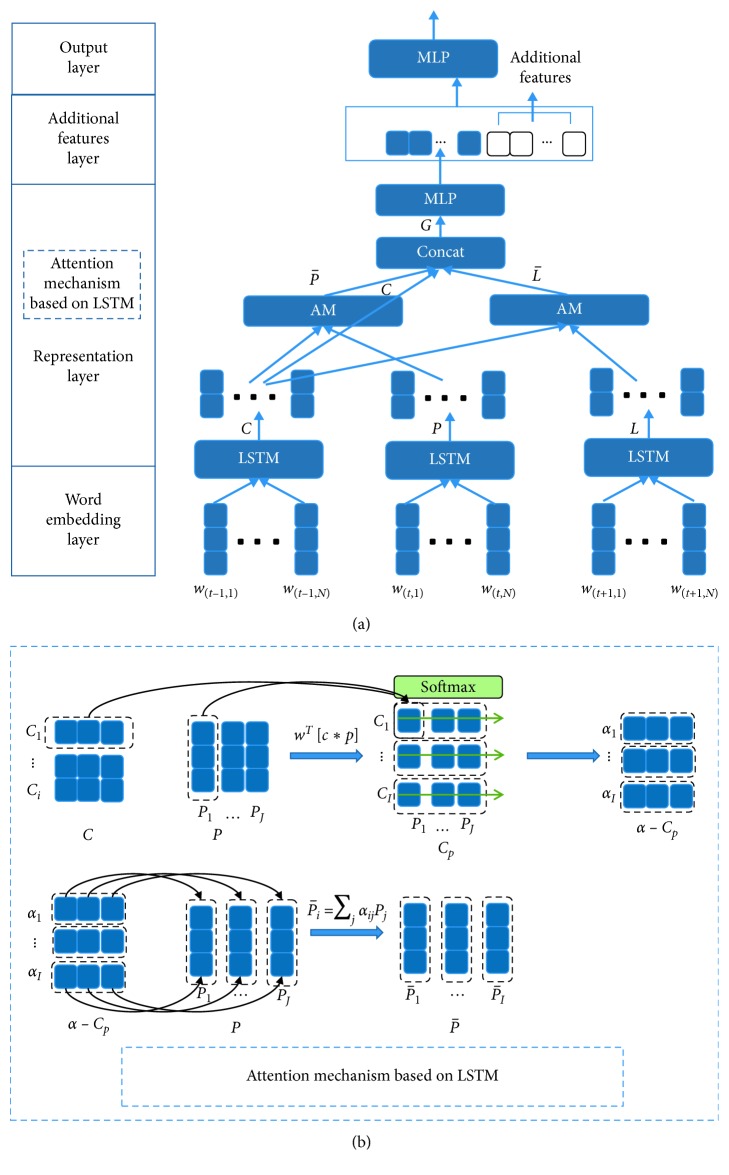
Text classification model with attention mechanism and additional features based on LSTM and consecutive sentences. (a) When the inputs are sequential sentences, the overall process of the text classification model is based on LSTM. The length of the sequential sentences is 3. (b) The detailed implementation of the AM module.

*(2) LSTM-Based Attention Mechanism*. To capture more information, the inputs of this module are the current utterance representation (*C* ∈ *R*^*I*×2*d*^) and the preceding (*P* ∈ *R*^*J*×2*d*^) or following utterance (*L* ∈ *R*^*K*×2*d*^) representation of *C*. Unlike calculating the contribution of each sentence to classification at the sentence level based on CNN, the main idea of BLSTM-based context-based attention mechanism proposed at the word level is to consider the importance distribution of each word in the contextual sentence relative to each word in the current sentence and update the contextual representation. Each word of a sentence corresponds to a context feature vector from its point of view. Finally, the final semantic feature vector can be learned by splicing it with the current sentence matrix. From this perspective, we first calculate attentions from current sentence to preceding sentence and then to following sentence separately in this module. The attention-based BLSTM, which will be discussed below, is derived from two similarity matrices, *M* ∈ *R*^*I*×*J*^ and *N* ∈ *R*^*I*×*K*^. *M*_*ij*_ indicates the similarity between the *i*-th word of current utterance and *j*-th word of preceding utterance, and *N*_*ik*_ indicates the similarity between the *i*-th word of current utterance and *k*-th word of following utterance. As shown in Figure 3(b), the similarity matrix is calculated by

(7) $$\begin{array}{c} M_{ij}=\varphi \left(C_{:i},P_{:j}\right),\\ N_{ik}=\varphi \left(C_{:i},L_{:k}\right), \end{array}$$

where *φ* is a function that encodes the similarity between its two input vectors, *C*_:*i*_ is the *i*-th column vector of *C*, *P*_:*j*_ is the *j*-th column vector of *P*, and *L*_:*k*_ is the *k*-th column vector of *L*. We choose *φ*(*c*, *p*)=*w*^*T*^[*c*^*T*^ · *p*] where *w* ∈ *R*^2*d*^ is a trainable weight vector and · is a matrix multiplication. Now, we use *M* and *N* to obtain the attentions and attended vectors from current sentence to preceding and following sentence to update the representations of context.

Current to preceding sentence attention signifies the importance distribution of each word in the preceding utterance for each word in current sentence. Let *α*_*i*_ ∈ *R*^*J*^ represent the attention weights on the words of preceding sentence by *i*-th word of current sentence, ∑_*j*_*α*_*ij*_=1 for all *i*. The attention weights are calculated by *α*_*i*_=softmax(*M*_*i*:_) ∈ *R*^*J*^, and subsequently, each attended representation of preceding sentence is ${\bar{P}}_{i}=\sum_{j}\alpha_{ij}P_{j}$ as shown in Figure 3(b) which is the overall information of the preceding utterance that is important for the *i*-th word of current utterance. Hence, $\bar{P}$ is a 2D-by-I matrix containing the attended preceding sentence vectors for the entire words of current utterance.

Same as current to preceding sentence attention, the attention of current to following utterance signifies which words of following sentence are most relevant to each word of current sentence. The attention weights are computed by *β*_*i*_ = softmax(*N*_*i*:_) ∈ *R*^*K*^, and finally each attended representation of following sentence by the *i*-th word of current sentence is ${\bar{L}}_{i}=\sum_{k}i_{ik}L_{k}$. Hence, $\bar{L}$ is a 2D-by-I matrix containing the attended following sentence vectors for the whole current sentence.

Finally, the current sentence matrix and the attention vectors which contain part of information of preceding and following sentence are combined together to yield *G*, in which each column vector can be considered as the contextual-aware representation of each word of current sentence. We define *G* by

(8) $$\begin{array}{c} G_{:i}=\left[C,\bar{P},\bar{L}\right]\in R^{6d\times I}, \end{array}$$

where *G*_:*i*_ is the *i*-th column vector. Then, we need to capture the interaction among the words of current sentence conditioned on the preceding and following sentence. We use two layers of bidirectional LSTM, with the output size of *d* for each direction. Finally, we obtain a vector from last column *μ* ∈ *R*^2*d*^ as the final representation of semantic features.

The difference between CNN and LSTM-based AM is that CNN-based attention uses a random vector as a reference to learn the weight of each sentence in the final representation; the LSTM-based attention uses the current sentence as the reference to update the representation of the preceding and following sentences and to retain the most important part.

### 3.3. Additional Features Layers

In the aspect of feature importance selection, a few features extracted by feature engineering in traditional text categorization methods have more advantages than those extracted by neural network. So, we also use some traditional methods to get some statistic values which can be represented as additional features concatenated with the CNN or LSTM feature representation. In this way, it is equivalent to separating the traditional feature selection from the training process of neural network and finally fusing the features of the two parts. This fusion method can not only give full play to the advantages of traditional feature construction method and neural network but also avoid the problem of reducing the generalization ability of the model by artificially setting the number of features in traditional feature construction method so that the text classification model can better and faster select the most meaningful features for text and avoid a large number of redundant features. We can also retain the original low-order features as well as utilize high-order features. The additional features consist mainly of the following parts:

1. The first part is the statistical features commonly used in traditional classification methods. In order to ensure the simplicity of the model, only the length of the text is used here. This paper argues that it can improve the performance of the model. For example, shorter sentence most likely is answer like “Uh yeah”. In this case, the length of the sentence 2 is a value of additional features.
2. The second part is the low-order feature obtained by feature selection, which only acts on the current sentences to be classified. We first use BOW to represent sentence and then obtain feature weights by TF-IDF. Finally, we reduce the dimension and extract the most relevant features by CHI [13], one method of feature selection. We compare using the statistical characteristics of words in training and testing to capture more information.
3. The third part is probability features, that is to say, a classifier different from this model is used to classify the current text, and the probability values of the classification results are taken as additional features. In this paper, a simple logistic regression (LR) model is used as a base classifier to obtain the probability distribution characteristics. For example, if the dataset has 10 categories, the probability distribution containing 10 values will be obtained after using the base classifier, which will be part of the additional features layer. The reason for this design is that we hope to use the idea of ensemble to enhance the diversity of models and reduce the possibility of overfitting by introducing other simple classifiers. This paper will analyze the applicability of this method in experiments.

Note that when predictions are used, we need to go through following procedure:

1. Train a logistic regression model and use it to generate predictions of the current sentence, for training data and test data.
2. Splicing the length of the current sentence with the value obtained by feature extracting and probabilities for adding additional features in the training set to train the models proposed in this paper.
3. Splicing the length of the current sentence with the value obtained by feature extracting and probabilities for adding additional features in test data and applying these new models.

Finally, we can get a vector *q* ∈ *R*^2*d*+*T*+*C*+1^ which is composed by the representation obtained from the model we proposed, the statistical features like TF-IDF, the probabilities, and the length of sentence, and *C* is the number of categories.

### 3.4. Output Layer

The output layer takes as input the sentence representation with additional features and outputs *z* ∈ *R*^*k*^:

(9) $$\begin{array}{c} z=\text{softmax}\left(Wq+b\right). \end{array}$$

The final output *z* represents the probability distribution over the set of *k* classes.

## 4. Data and Experimental Setup

In this section, we conduct experiments and demonstrate that our models can improve the performance of text classification. We first introduce the experimental dataset, and then we describe the parameter settings determined by cross validation in our experiments. Finally, we compare our results with other works.

### 4.1. Data

We evaluate our model on the DA classification task using the following datasets:

- SWDA (Switchboard Dialog Act Corpus [16]): a dialog corpus of 2-speaker conversations
- MRDA (ICSI Meeting Recorder Dialog Act Corpus [37]): a dialog corpus of multiparty meetings

These sets have been widely used in the community for DA classification. Train, validation, and test splits on the set were taken as defined in [36]; summary statistics are shown in Table 1.

### 4.2. Results and Discussion

For all the models, most of the experimental parameters were chosen based on the literature or our experience with other DNN-based text classification tasks [28, 29, 38]. We select the best parameters using the grid search. In all experiments, we use pertained embeddings to initialize the embedding layer. Besides, we apply dropout [39] to the word embeddings. AdaGrad is exploited as the optimizer in our experiments, and our best results were obtained with three contextual utterances for SWDA and MRDA.

In all the result tables, we use accuracy (%) and epoch time (s) as evaluation indicators. We use the symbol “/” to isolate the results of different datasets. The left side of the symbol represents SWDA's result, and the right side represents MRDA's result.

#### 4.2.1. Baseline Models

We define two models as baseline, which are shown in Figure 1: one is based on CNN and the other one is based on BLSTM. Also, the input for both of them is a single utterance a time without any contextual information.  Table 2 presents our hyperparameter choices.

Table 3 shows the classification results on both datasets when no context information, no attention, and no additional features are used. We can see that when no pretrained embeddings are used, there is a performance degradation of 1–2.5% for both datasets. Besides, when there is no contextual information, the CNN model outperforms the BLSTM model.

#### 4.2.2. CNN/BLSTM + Attention Mechanism

As described in Section 3.2, the method to incorporate context information is to use the attention mechanism to act on the preceding and current and following utterance at the representation layer based on CNN or BLSTM. For the two models, we keep the same model parameters with the basic model parts apart from the learning rate, which should be changed to 0.1 demonstrated by grid search.  Table 4 summarizes the results of the models using contextual information and attention mechanism. We have the following observations: (1) Using contextual information for DA classification is quite effective, and both models significantly outperform the baselines. This proves the validity of introducing context information to provide context for semantic understanding. (2) Both contextual information and AM can greatly improve the BLSTM-based model. However, in the CNN-based model, AM plays a relatively small role. That means, under this context, two kinds of AMs are designed in this paper; the one based on BLSTM is more effective. (3) Although the accuracy based on CNN is relatively low, it has great advantages in time efficiency.

When based on CNN, as described in Section 3.2, a simple concatenation or average value of context achieves similar performance in our experiments, and this is because these methods are equivalent to taking the same weights for all utterances. However, using AM to learn the different weights over three utterances can learn which utterance is more meaningful so that the result can improve accuracy by 0.3% on SWDA and 0.6% on MRDA. Moreover, because of the simplicity of attention calculation, it has more prominent advantages in time efficiency.

When introducing contextual sentences, BLSTM-based models are generally better than CNN-based models in both datasets, which shows that for multiple continuous sentences, the vector obtained by fusing the output of multiple BLSTM encoders can well describe the semantic relationship between sentences. In addition, our results based on BLSTM are consistently improved after introducing attention to capture the useful information from the preceding and following utterance and then adding them to the current utterance described in Figure 3. This fully illustrates that this attention method, which starts from the word vectors of the current sentences to be classified, calculates the most relevant contextual content representation of each word and fuses them according to the weight of the words and can effectively filter useless word information while introducing context-related semantic information. This attention mechanism can enhance the influence of keyword information in context on classification results, thus effectively alleviating the problem that invalid word information has certain influence on classification results and significantly improve the effect of text classification.

From the epoch time required for each model, the experimental time of the CNN-based model is usually shorter. This is because each BLSTM encoder learns more parameters and the calculation process of attention mechanism based on BLSTM is complicated. Therefore, the attention mechanism based on CNN fusing context information can improve the effect of the model to a certain extent and has higher efficiency; the attention mechanism based on BLSTM can improve the effect of classification model greatly, but it sacrifices certain efficiency due to the complexity of calculation.

#### 4.2.3. CNN/LSTM + Additional Features

As described in Section 3.3, another method to get better representation is to extract some additional features such as the length of utterance, the TF-IDF values, and the probabilities.  Table 5 shows the results for different setups in CNN and BLSTM models to evaluate the impact of different features.

From Table 5 we can see that using additional features to model representation for DA classification is effective and both models significantly outperform the basic models on both datasets. It also shows that in terms of the attended representation, using statistical features (length, TF-IDF) and probabilistic features separately achieves similar performance. This shows that the traditional feature selection method and the probability features can make up for the more important features that cannot be captured by deep learning and can play a certain auxiliary role in the overall classification model. Although using additional features is not as much improved as the AM, it is still effective. The reason is that after the AM are obtained more useful features, and the original statistical features only play a supporting role.

From the iteration time, it can be seen that the introduction of different feature combinations will not have a significant impact on the overall time efficiency, which fully proves the effectiveness and efficiency of the feature fusion method proposed in this paper.

#### 4.2.4. Comparison with Other Works

Table 6 compares our results with other works. To the best of our knowledge, [38] is the newest research in DA classification, that is, CR-attention model in Table 6. In that research, a model based on two-layer LSTM and attention is proposed. Overall, our model shows much better results no matter from accuracy or epoch time. While we train the model based on LSTM, our best model yields comparable results to the state of the art, 73.9% against 73.8% on SWDA and 85.9% against 84.3% on MRDA. Also, the state-of-the-art model is more complicated than our best model, which needs longer time because it first uses LSTM to get each representation of utterance, then uses LSTM to obtain advanced features, and finally utilizes AM to learn the final representation. Obviously, that model needs more features to learn, and its training speed is obviously slower than ours. And our model is multimodule combination; if you want the model to be simpler, we can remove the additional features module; after all, our model still can achieve better results, 73.6% on SWDA and 85.4% on MRDA, only using LSTM + attention. So, our model is more scalable and effective.

## 5. Conclusion

In this paper, we explore several approaches to incorporate context information in the deep learning framework for DA classification, including expanding designing a kind of attention-based vector for CNN as well as a kind of attention mechanism-based matrix for BLSTM, using sentence length, probabilities, and TF-IDF value as additional features on the represent layer. Compared to the baseline, using CNN or BLSTM, when input is one utterance, our models can effectively leverage the context information and the raw features to achieve significantly better performance. The proposed classification model based on BLSTM that uses the matrix to calculate the attention values and updates the sentence representation is very effective and novel. Furthermore, our results represent the state of the art for DA classification on the SWDA and MRDA datasets in terms of time and accuracy. This article shed lights on the use of AMs and context information for similar tasks.

We believe that future research could focus on the introduction of other networks such as GRU to improve our representation. We also will explore to design other AMs to improve accuracy and explore more datasets to improve our models.

## Figures and Tables

**Table 1:** Dataset summary statistics.

Dataset	*C*	*V* (k)	Train (k)	Validation (k)	Test (k)
SWDA	43	20	193	23	5
MRDA	5	12	78	16	15

*C* is the number of classes; *V* is the vocabulary size. The train/validation/test indicates the number of utterances in all dialogs.

**Table 2:** Experiment values and choices of some hyperparameters.

Hyperparameter	Choice	Experiment values
Learning rate	0.01	0.1, 0.01, 0.001
LSTM output dimension	100	50, 100, 150
LSTM direction	Bidir	Unidir, bidir
LSTM pooling	Last	Mean, last
CNN filter numbers	100	100, 300, 500, 700
CNN filter height	3	1, 2, 3, 4, 5
Word vector dimension	200	100, 200, 300

**Table 3:** Classification accuracy (%) and epoch time (s) when using the basic models based on CNN and BLSTM.

Models	Accuracy	Time
CNN with no pretrained embeddings	68.9/75.4	121/100
CNN with pretrained embeddings	**71.1/78.3**	110/89
BLSTM with no pretrained embeddings	68.2/75.0	587/450
BLSTM with pretrained embeddings	70.6/77.1	571/423

**Table 4:** Results of models with different context process methods.

Models	Accuracy	Time
CNN with contextual info	72.3/81.8	310/260
CNN with contextual info and AM	72.6/82.4	346/287
LSTM with contextual info	72.5/82.1	923/700
LSTM with contextual info and AM	**73.6/85.4**	1050/794

**Table 5:** Results of models when using additional features by different ways.

Models	Accuracy	Time
Attended representation with length + TF-IDF (CNN)	72.7/83.1	379/310
Attended representation with probs (CNN)	72.8/83.0	396/321
Attended representation with probs + length + TF-IDF (CNN)	73.0/83.4	412/335
Attended representation with length + TF-IDF (BLSTM)	73.7/85.6	1084/905
Attended representation with probs (BLSTM)	73.6/85.7	1107/918
Attended representation with probs + length + TF-IDF (BLSTM)	**73.9/85.9**	1141/924

**Table 6:** Results of our models and other methods from the literature: HMM [16]; CNN-FF and LSTM-FF [36]; CA-LSTM: contextual attentive LSTM [40]; CR-attention: CNN + RNN + attention [38].

Models	Accuracy	Time
Our best model based on CNN	73.0/83.4	412/335
Our best model based on LSTM	**73.9/85.9**	1141/924
CR-attention	73.8/84.3	1320/1182
CNN + FF	73.1/84.6	732/620
LSTM + FF	69.6/84.3	940/762
HMM	71.0/—	210/—
CA-LSTM	72.6/—	1026/—

## Data Availability

The SWDA data used to support the findings of this study have been deposited in http://compprag.christopherpotts.net/swda.html.
